# Clinicopathological significance of cancer stem cells marked by CD133 and KAI1/CD82 expression in laryngeal squamous cell carcinoma

**DOI:** 10.1186/1477-7819-12-118

**Published:** 2014-04-24

**Authors:** Lan Yu, Lei Zhou, Shiwu Wu, Xiaomeng Gong, Zhenzhong Feng, Li Ma, Bo Zhu, Nan Yao, Danna Wang, Huiming Dong

**Affiliations:** 1 Department of Pathology, the First Hospital Affiliated to Bengbu Medical College, Bengbu Medical College, 800 Zhihuai Ave, Longzihu, Bengbu, Anhui, China; 2 Department of Surgical Oncology, the First Hospital Affiliated to Bengbu Medical College Anhui Province, Bengbu Medical College, 800 Zhihuai Ave, Longzihu, Bengbu, Anhui, China

## Abstract

**Background:**

Presently, CD133 is one of the hottest markers to characterize cancer stem cells and KAI1/CD82 is reported as an important marker for the metastasis and prognosis of many cancers. The purpose of our study is to explore the relationship between cancer stem cells (CSCs) marked by CD133 and KAI1/CD82 expression and the clinicopathological characteristics of patients with laryngeal squamous cell carcinoma (LSCC).

**Methods:**

Immunohistochemical analysis was used to detect the expression of CD133 and KAI1/CD82 in 83 archival surgical specimens of human LSCC and 83 cases of normal laryngeal tissues.

**Results:**

In LSCC, positive rates of 49.4% and 41.0% were obtained for CD133 and KAI1/CD82, respectively. The expression of CD133 in LSCC tissues was significantly higher than that in normal tissues (*P* < 0.001), and the expression of CD133 was positively associated with pTNM stage (*P* = 0.005), pathological grade (*P* = 0.001), and lymph node metastasis (*P* < 0.001). The reduced expression of KAI1/CD82 was present in LSCC tissues. The positive rate of KAI1/CD82 expression was negatively correlated with pTNM stage (*P* = 0.014), pathological grade (*P* < 0.001), and lymph node metastasis (*P* = 0.007). A correlation analysis showed that there was a negative relationship between the expression of CD133 and KAI1/CD82 protein in LSCC tissues (*P* < 0.001). By Kaplan-Meier analysis, the expression of CD133 was negatively correlated with overall survival (OS) (log-rank = 40.949, *P* < 0.001) and disease-free survival (DFS) (log-rank = 39.307, *P* < 0.001) time of LSCC. The expression of KAI1/CD82 was positively correlated with OS (log-rank = 40.279, *P* < 0.001) and DFS (log-rank = 39.271, *P* < 0.001) time of LSCC. Cox regression analysis: the expression of CD133 and KAI1/CD82, and pTNM stages were independent prognostic factors of LSCC (*P* < 0.05).

**Conclusions:**

Thus the detection of CD133 and KAI1/CD82 proteins may be used as a potential indicator of LSCC prognosis.

## Background

In the United States, laryngeal carcinoma accounted for approximately 0.82% of new cancer diagnoses and 0.40% of all cancer deaths in 2012 [1]. The major pathological type of laryngeal cancers is squamous cell carcinoma, accounting for 99% of laryngeal malignant tumors. Although rapid progress has recently been made in treatment, the prognosis for patients with laryngeal squamous cell carcinoma (LSCC) remains unsatisfactory. A major problem in finding treatments is the frequent resistance to drugs which emerges. This is linked to the development and maintenance of a small population of tumor cells, termed cancer stem cells (CSCs). These cells have the properties of self-renewal, proliferation, and multilineage differentiation and are responsible for sustaining the tumor [2] and are also thought to initiate tumor metastasis and therapy-resistance [3,4]. A commonly investigated potential CSC marker is CD133 (also known as prominin-1), a 120 kDa five transmembrane domain cell surface glycoprotein, which was initially considered to be one marker of hematopoietic stem cells [5,6]. Now, CD133 may represent a putative cancer stem cell marker in many solid tumors, such as human colon cancer [7,8], breast cancer [9,10], gastric cancer [11,12], glioblastoma [13], lung cancer [14,15], liver cancer [16,17], pancreatic cancer [18], prostate cancer [19], and cholangiocarcinoma [20].

Cancer metastasis involves multiple steps with a high degree of complexity and requires the contribution of a variety of molecules. The *KAI1/CD82* gene was originally identified as a suppressor of metastasis of tumor in prostate carcinoma [21]. Recent study has shown that *KAI1/CD82* gene expression is under-regulated in most metastatic cancers [22]. It is a member of the tetraspan transmembrane superfamily (*TM4SF*) and is a gene located on human chromosome 11p11.2. KAI1/CD82 plays an important role in cell fusion, adhesion, migration, signaling, fertilization, differentiation, and invasion [22-26]. Decreased KAI1/CD82 expression has been observed to correlate with metastasis and poor prognosis in many human solid tumors, such as prostate cancer [27], lung cancer [28], breast cancer [29], colon cancer [30], gastric cancer [31], liver cancer [32], and kidney cancer [33].

To date, the correlation between CD133 and KAI1/CD82 expression in LSCC is unknown. Therefore, this study intends to investigate CD133 and KAI1/CD82 expression in the specimens of postoperative LSCC patients following primary laryngeal resection in order to determine the correlation between the expression of CD133 and KAI1/CD82 and clinicopathological characteristics acceptable.

## Methods

### Patients and specimens

Paraffin embedded sections of 83 LSCCs and 83 normal laryngeal tissues were obtained from the Department of Pathology, the First Hospital Affiliated to Bengbu Medical College from January to November 2003. We excluded patients who received preoperative chemotherapy or radiotherapy. Approval for this study was not required by the ethical committee because the experiments carried out did not relate to patient privacy, impairment or treatment. The age of the patients ranged from 43 to 84 years; the median age was 62.1 years. The patients consisted of 75 males and 8 females. There were 43 cases whose tumors were < 2.0 cm in diameter and 40 cases whose tumors were ≥ 2.0 cm. Thirty were at grade I, 47 were at grade II, and 6 were at grade III, according to the grading system of the World Health Organization. Fifty-one were of supraglottic type, 29 were glottic type, and three were subglottic type. A total of 49 specimens had no lymph node metastasis, whereas 34 specimens showed lymph node metastasis. According to clinical staging of pTNM, 21 were stage I, 32 were stage II, 22 were stage III, and 8 were stage IV.

### Immunohistochemical analysis

All samples were fixed in 10% buffered formalin and embedded in paraffin. Four- micrometer thick tissue sections were used for analysis. All sections were deparaffinized and dehydrated with graded alcohol. Then, the sections were washed for ten minutes in PBS at pH 7.2. The endogenous peroxidase activity was quenched by incubation in methanol containing 3% H_2_O_2_ for ten minutes at room temperature, then heated for 30 minutes at 95°C to repair the antigens and finally rinsed in PBS. After several washes in PBS, sections were blocked with goat serum for 20 minutes at room temperature, and then incubated with mouse monoclonal CD133 (Santa Cruz Biotechnology Inc., Santa Cruz, CA, USA) and KAI1/CD82 (Santa Cruz Biotechnology Inc., Santa Cruz, CA, USA) primary antibodies overnight at 4°C in a humidified chamber. The slides were treated with polymer enhancer (reagent A) for 20 minutes at room temperature. Washing in PBS, the slides were treated with goat anti-mouse antibody (reagent B) for 30 minutes at room temperature. After a complete wash in PBS, the slides were developed in freshly prepared diaminobenzidine (DAB) solution for eight minutes, and then counterstained with hematoxylin, dehydrated, air-dried, and mounted.

Serial sections of LSCC were run in parallel with the primary antibody replaced by PBS and rabbit IgG1 as blank and negative controls.

### Evaluation of score

Slides were reviewed independently by two observers to evaluate the staining pattern of the protein under the light microscope. Ten visual fields were randomly selected from each slide. In scoring expression of CD133 and KAI1/CD82 proteins, both the extent and intensity of immunopositivity were considered. The intensity of the positive result was scored as follows: 0, negative; 1, weak; 2, moderate; 3, strong. The extent of positivity was scored according to the percentage of cells that stained positive: < 10% is 1; 11 to 50% is 2; 51 to 75% is 3; > 75% is 4. The final score was determined by multiplying the intensity of positivity and the extent of positivity scores, which yielded a range from 0 to 12. Expression of CD133 and KAI1/CD82 were considered positive when the scores were ≥ 3.

The positive expression of CD133 was found mainly on the membrane and cytoplasm of LSCC cells and normal laryngeal tissues. The positive expression of KAI1/CD82 was found mainly on the membrane and cytoplasm of LSCC cells and normal laryngeal tissues. They were presented as a brown granular material.

### Statistical analysis

Fisher’s exact test, Pearson Chi-square test for trends in proportions, Spearman’s correlate analysis, and Kaplan-Meier’s method with log-rank test or Cox regression method for univariate or multivariate OS analysis were used to assess the associations among the positive staining of CD133 or KAI1/CD82 and clinicopathological indices. SPSS 17.0 software for windows (Chicago, IL, USA) was used for this purpose. A value of *P* < 0.05 was considered statistically significant.

## Results

### The association between the expression of CD133 or KAI1/CD82 and clinicopathological factors

**Figure 1:**
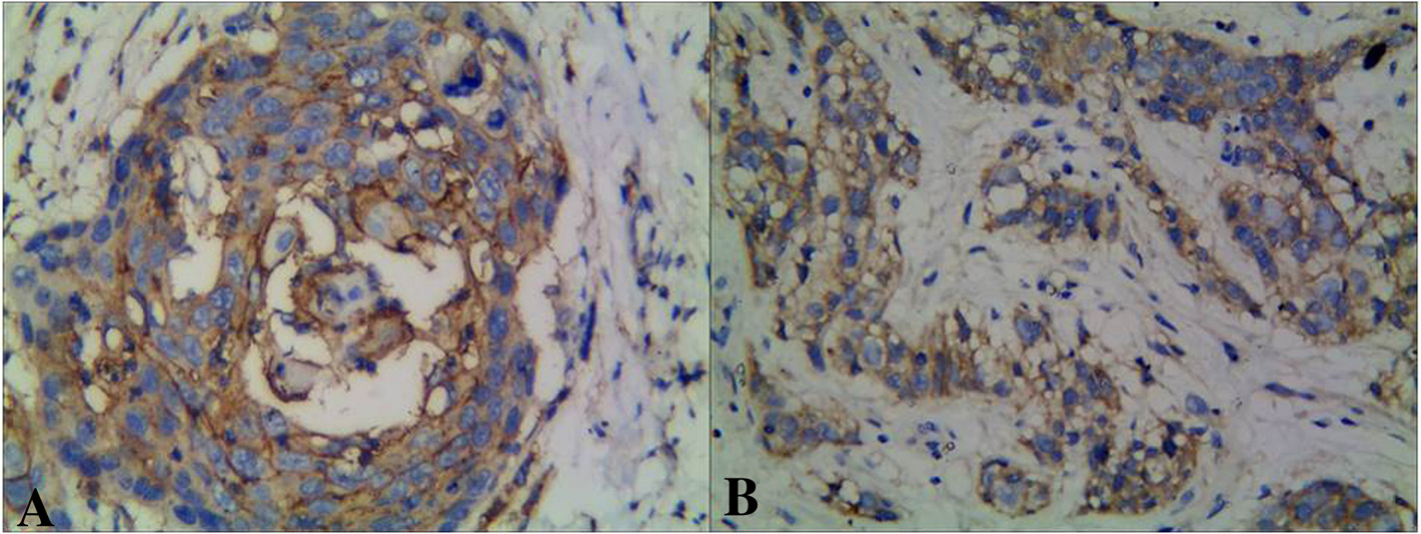
**Expression of CD133 protein in laryngeal squamous cell carcinoma. (A)** CD133 was expressed as positive in the membrane of cancer cells (CD133 × 400). **(B)** CD133 was expressed as positive in the membrane of cancer cells at the invasive cancer (CD133 × 400).

**Figure 2:**
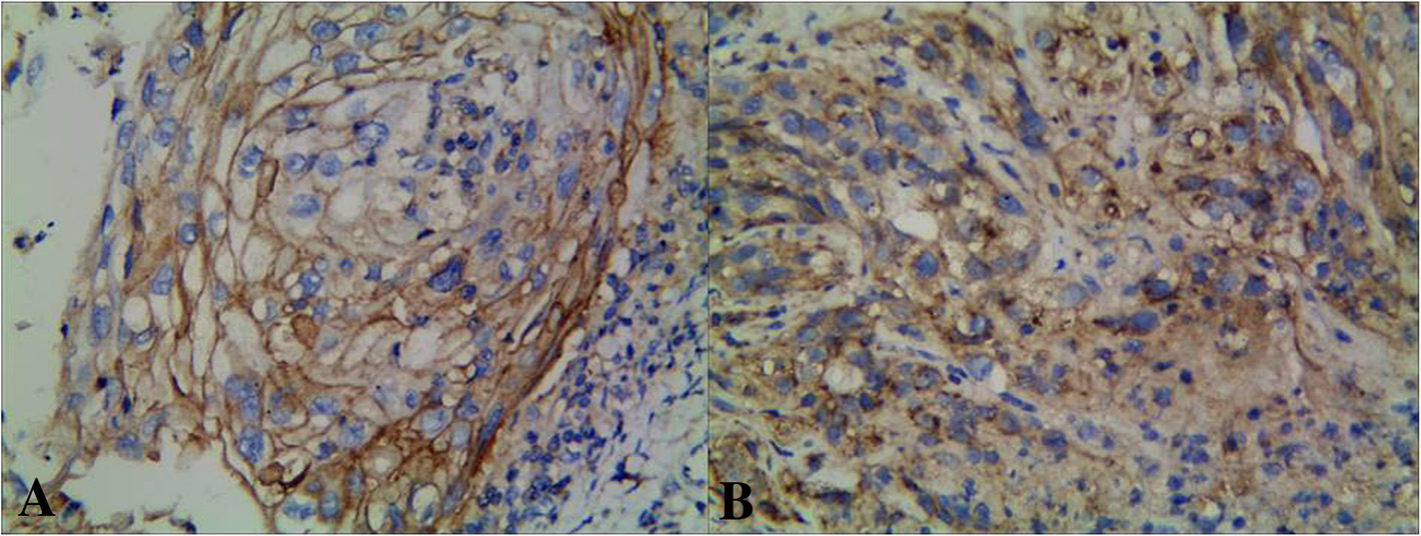
**Expression of KAI1/CD82 protein in laryngeal squamous cell carcinoma. (A)** KAI1/CD82 was expressed as positive in the membrane of cancer cells (KAI1/CD82 × 400). **(B)** KAI1/CD82 was expressed as positive in membrane and cytoplasm of cancer cells (KAI1/CD82 × 400).

CD133 protein was expressed positively in 49.4% (41/83) of LSCC and 4.8% (4/83) of normal laryngeal tissues. There was a significant difference between the LSCC group and the normal laryngeal tissues (*P* < 0.01) (Figure 1A and B). There was a positive relationship between the expression of CD133 and histological grade, pTNM stage, and lymph node metastasis (*P* < 0.05). The positive expression of KAI1/CD82 was 41.0% (34/83) in the LSCC group and 96.4% (80/83) in normal laryngeal tissues. A significant difference was found between the LSCC group and the normal group (*P* < 0.05) (Figure 2A and B). There was a negative relationship between the expression of KAI1/CD82 protein and alcohol, histological grade, pTNM stage, and lymph node metastasis (*P* < 0.05). However, the expression of CD133 and KAI1/CD82 was not associated with gender, age, tumor localization, smoking, and tumor diameter (*P* > 0.05) (Table 1).

**Table 1:** Correlation of CD133 and KAI1/CD82 expression to clinicopathogical characteristics in laryngeal squamous cell carcinoma (LSCC)

**Variable**	**CD133**		***P*-value**	**KAI1/CD82**		***P*-value**
	**Negative**	**Positive**		**Negative**	**Positive**	
Gender			> 0.05			> 0.05
Male	36	39		46	29	
Female	6	2		3	5	
Age			> 0.05			> 0.05
< 60	15	12		15	12	
≥ 60	27	29		35	22	
Location			> 0.05			> 0.05
Supraglottic	26	25		26	25	
Glottic	15	14		20	9	
Subglottic	1	2		3	0	
Diameter of tumor			> 0.05			> 0.05
< 2.0 cm	23	20		22	21	
≥ 2.0 cm	19	21		27	13	
Smoking			> 0.05			> 0.05
No	18	13		15	16	
Yes	24	28		34	18	
Alcohol			> 0.05			< 0.05
No	20	13		14	19	
Yes	22	28		35	15	
Grade of tumor			< 0.01			< 0.001
Well-differentiated	23	7		9	21	
Moderately-differentiated	18	29		35	12	
Poorly-differentiated	1	5		5	1	
Lymph node metastasis			< 0.001			< 0.01
No	33	16		23	26	
Yes	9	25		26	8	
pTNM stage			< 0.05			< 0.05
I and II	33	20		26	27	
III and IV	9	21		23	7	

### Prognosis and multivariate analysis

**Figure 3:**
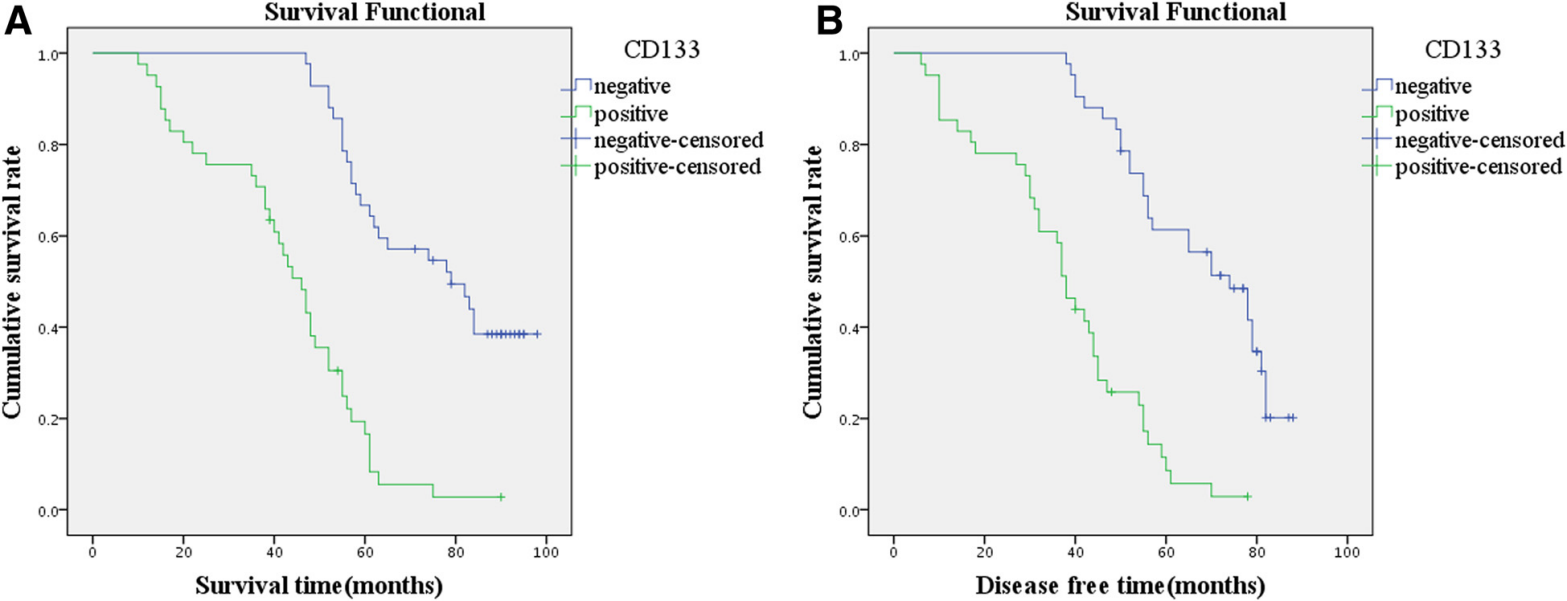
**Kaplan-Meier survival analysis by CD133 status (n = 83). (A)** The green line represents the CD133-positive group patients with a trend of worse survival than the blue line representing CD133-negative group patients (log-rank = 40.949, *P* = 0.000). Mean overall survival (OS) time was 42.4 months for the CD133-positive group and 73.3 months for the CD133-negative group. **(B)** The green line represents CD133-positive group patients with a trend of worse survival than the blue line representing CD133-negative group patients (log-rank = 39.307, *P* = 0.000). Mean DFS for patients was 37.1 months for the CD133-positive group and 65.6 months for the CD133-negative group.

**Figure 4:**
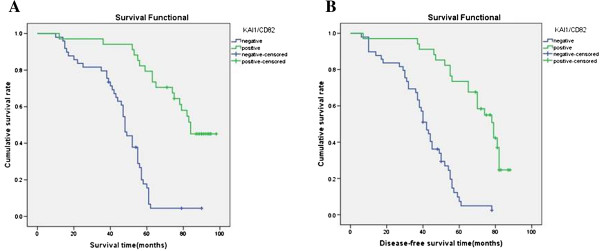
**Kaplan-Meier survival analysis by KAI1/CD82 status (n = 83). (A)** The green line represents the KAI1/CD82-positive patient group with a trend of better survival than the blue line representing the KAI1/CD82-negative patient group (log-rank = 40.279, *P* = 0.000). Mean overall survival (OS) time was 76.0 months for the KAI1/CD82-positive group and 45.5 months for the KAI1/CD82-negative group. **(B)** The green line represents the KAI1/CD82-positive patient group with a trend of better survival than the blue line representing the KAI1/CD82-negative group patients (log-rank = 39.271, *P* = 0.000). Mean DFS patients was 68.0 months for the KAI1/CD82-positive group and 40.1 months for the KAI1/CD82-negative group.

Follow-up data showed that the OS rate and DFS rate for CD133-positive patients were significantly poorer than that of CD133-negative patients (*P* < 0.001, *P* < 0.001, Figure 3). Also, there was a significantly increasing trend in the mean OS survival time and DFS time between the carcinomas with the expression of KAI1/CD82 and those without (*P* < 0.001, *P* < 0.001, Figure 4). In the 83 LSCC patients, a univariate analysis (Table 2) revealed that the DFS survival significantly correlated with expression of CD133 (*P* < 0.001) and KAI1/CD82 (*P* < 0.001), tumor location *(P* = 0.033), grade of tumor (*P* = 0.002), lymph node metastasis (*P* = 0.004), and pTNM stages (*P* < 0.001). A multivariate analysis revealed expression of CD133 and KAI1/CD82, lymph node metastasis, and pTNM stages were independent prognostic factors for DFS and OS (*P* < 0.05) (Tables 3 and 4).

**Table 2:** Results of univariate analyses of disease-free survival (DFS) and overall survival (OS) time

**Variable**	**n**	**Mean DFS**	***P*-value**	**Mean OS**	***P*-value**
		**(months)**		**(months)**	
CD133			< 0.001		< 0.001
+	41	42.4 ± 18.2		37.1 ± 17.7	
-	42	73.3 ± 16.7		65.6 ± 15.4	
KAI1/CD82			< 0.001		< 0.001
+	34	76.0 ± 19.3		68.0 ± 17.5	
-	49	45.5 ± 16.8		40.1 ± 16.7	
Gender			0.035		0.067
Male	75	56.4 ± 23.0		50.0 ± 21.9	
Female	8	73.1 ± 21.9		65.6 ± 17.0	
Age			0.854		0.280
< 60	27	58.2 ± 21.5		50.9 ± 19.5	
≥ 60	56	57.9 ± 24.3		51.8 ± 23.0	
Location			0.004		0.065
Supraglottic	51	60.3 ± 24.1		53.9 ± 22.5	
Glottic	29	56.4 ± 21.8		49.6 ± 20.5	
Subglottic	3	35.0 ± 13.7		29.3 ± 11.2	
Diameter of tumor			0.015		0.217
< 2.0 cm	43	65.5 ± 19.5		58.5 ± 18.5	
≥ 2.0 cm	40	50.0 ± 24.6		44.0 ± 22.9	
Smoking			0.122		0.132
No	31	62.0 ± 26.4		55.3 ± 22.4	
Yes	52	55.6 ± 21.2		49.3 ± 20.1	
Alcohol			0.365		0.163
No	33	62.4 ± 24.1		54.9 ± 22.5	
Yes	50	55.1 ± 22.6		49.2 ± 21.3	
Grade of tumor			< 0.001		0.009
Well-differentiated	30	71.7 ± 18.7		64.4 ± 17.6	
Moderately-differentiated	47	50.4 ± 22.1		44.5 ± 20.7	
Poorly-differentiated	6	49.3 ± 25.3		42.2 ± 22.8	
Lymph node metastasis			< 0.001		0.003
No	49	67.6 ± 18.9		60.5 ± 17.4	
Yes	34	44.2 ± 22.4		38.6 ± 21.3	
pTNM			< 0.001		< 0.001
I and II	53	68.3 ± 19.2		61.3 ± 17.4	
III and IV	30	40.0 ± 18.4		34.3 ± 18.0	

**Table 3:** Results of multivariate analyses of disease-free survival (DFS) time

**Covariate**	**B**	**SE**	**Sig**	**Exp(B)**	**95%CI**
CD133	0.747	0.354	0.035	2.110	1.055 to 4.221
KAI1/CD82	-1.282	0.432	0.003	0.278	0.119 to 0.647
pTNM	1.203	0.350	0.001	3.331	1.678 to 6.611
Lymph node metastasis	0.643	0.291	0.027	1.903	1.075 to 3.366

**Table 4:** Results of multivariate analyses of overall survival (OS) time

**Covariate**	**B**	**SE**	**Sig**	**Exp(B)**	**95%CI**
CD133	0.734	0.351	0.037	2.083	1.046 to 4.146
KAI1/CD82	-1.486	0.449	0.001	0.226	0.094 to 0.545
pTNM	1.191	0.349	0.001	3.292	1.659 to 6.530
Lymph node metastasis	0.747	0.287	0.009	2.110	1.203 to 3.701

### Correlation of CD133 and KAI1/CD82 in LSCC

There was a negative correlation between CD133 expression and KAI1/CD82 expression in LSCC (r = -0.578, *P* < 0.001).

## Discussion

Cancer stem cells (CSCs), also known as tumor initiating cells (TICs), are a subpopulation of tumor cells with the stem cell capacity for self-renewal. They give rise to the differentiated cells, and generate the heterogeneous lineages of cancer cells that comprise the bulk of the tumor [2,7,11,20,34-39]. CSCs may originate by malignant transformation of normal stem cells or arise from restricted progenitors of more differentiated cells [40]. The protein CD133 is one of the hot CSC markers in a variety of tumors [7-20]. In this study, we found that the positive expression of CD133 was 49.4% in LSCC patients. The study investigated the expression of CD133 protein in 83 LSCC specimens with follow-up data and the results indicated that CD133 protein expression level was positively correlative with grade of tumor (*P* = 0.001), lymph node metastasis (*P* < 0.001), and pTNM stage (*P* = 0.005). Furthermore, we also found the expression of CD133 was significantly associated with OS time (*P* < 0.001) and DFS time (*P* < 0.001), suggesting the expression of CD133 might be a potential prognostic factor in LSCC. Another result is that not only LSCC cell express CD133 but also normal laryngeal tissue (squamous epithelial cells). This indicated that CD133 might play an important role in tumorigenesis [8].

The metastasis suppressor gene *KAI1/CD82* might be a useful marker for the metastatic and prognostic potential in a series of human tumors. The precise mechanism for the regulation of *KAI1/CD82* is unclear. But, in the progression of many tumors, the most common mechanism was down-regulation or loss-regulation rather than mutation [22,24]. Our study results showed that *KAI1/CD82* was negatively associated with grade of tumors (*P* < 0.001), lymph node metastasis (*P* = 0.007), and pTNM stage (*P* = 0.014). From further research, we found that the *KAI/CD82*-positive-group correlated with longer survival time, a result which was in agreement with previous reports [32,41,42].

Our research also showed that CD133 expression was negatively correlated with KAI1/CD82 expression (*P* < 0.001). CSCs can manipulate stromal cells to their needs in distant organs and thus prime the foreign soil for their arrival by inducing a premetastatic niche [43]. This indicates that CSCs have high migratory potential and may be responsible for metastasis [44]. Down-regulation of *KAI1/CD82* may further promote the metastatic ability of CSCs. Although the precise molecular mechanism involved in this process is unclear, our research has potential clinical benefits. CD133 and *KAI1/CD82* expression, which could be detected by immunohistochemistry, might be a useful molecular marker to predict the prognosis in LSCC patients. It is concluded that the expression of CD133 and *KAI1/CD82* protein could be correlated with lymph node metastasis, grade of tumor, and pTNM stage in LSCC, and also concluded that they are useful prognostic factors for OS and DFS in LSCC. The combined detection of CD133 and *KAI1/CD82* can, to some extent, reflect the biological behavior of LSCC, thus giving the choice of molecular targeting therapy. However, the number of specimens in our study was relatively small. Further studies with larger sized specimens and molecular experiments are needed to verify the present observations.

## Conclusions

It is suggested that CD133 and *KAI1/CD82* may play an important role in the evolution of LSCC. And CD133 and *KAI1/CD82* should be considered as potential marker for the prognosis in patients with LSCC.

## Abbreviations

LSCC: laryngeal squamous cell carcinoma; CSC: cancer stem cells; pTNM: pathological tumor-node-metastasis; OS: overall survival; DFS: disease free survival; TM4SF: tetraspan transmembrane superfamily; DAB: diaminobenzidine.

## Competing interests

The authors declare that they have no competing interests.

## Authors’ contributions

YL, ZL and WSW carried out the design, analysis of pathology and drafted the manuscript. GXM, FZZ, ML, ZB and YN carried out sample collections and coordination. WDN and DHM performed the immunohistochemical staining. All authors read and approved the manuscript.
